# Force Mapping during the Formation and Maturation of Cell Adhesion Sites with Multiple Optical Tweezers

**DOI:** 10.1371/journal.pone.0054850

**Published:** 2013-01-25

**Authors:** Melanie Schwingel, Martin Bastmeyer

**Affiliations:** 1 Karlsruhe Institute of Technology (KIT), Zoological Institute, Cell- and Neurobiology, Karlsruhe, Germany; 2 DFG-Center for Functional Nanostructures (CFN), Karlsruhe Institute of Technology (KIT), Karlsruhe, Germany; University of Heidelberg Medical School, Germany

## Abstract

Focal contacts act as mechanosensors allowing cells to respond to their biomechanical environment. Force transmission through newly formed contact sites is a highly dynamic process requiring a stable link between the intracellular cytoskeleton and the extracellular environment. To simultaneously investigate cellular traction forces in several individual maturing adhesion sites within the same cell, we established a custom-built multiple trap optical tweezers setup. Beads functionalized with fibronectin or RGD-peptides were placed onto the apical surface of a cell and trapped with a maximum force of 160 pN. Cells form adhesion contacts around the beads as demonstrated by vinculin accumulation and start to apply traction forces after 30 seconds. Force transmission was found to strongly depend on bead size, surface density of integrin ligands and bead location on the cell surface. Highest traction forces were measured for beads positioned on the leading edge. For mouse embryonic fibroblasts, traction forces acting on single beads are in the range of 80 pN after 5 minutes. If two beads were positioned parallel to the leading edge and with a center-to-center distance less than 10 µm, traction forces acting on single beads were reduced by 40%. This indicates a spatial and temporal coordination of force development in closely related adhesion sites. We also used our setup to compare traction forces, retrograde transport velocities, and migration velocities between two cell lines (mouse melanoma and fibroblasts) and primary chick fibroblasts. We find that maximal force development differs considerably between the three cell types with the primary cells being the strongest. In addition, we observe a linear relation between force and retrograde transport velocity: a high retrograde transport velocity is associated with strong cellular traction forces. In contrast, migration velocity is inversely related to traction forces and retrograde transport velocity.

## Introduction

Cells exert forces onto their growth substrate during spreading and migration by forming adhesive contacts that connect the cellular cytoskeleton with the surrounding extracellular matrix (ECM). Force sensing and transmission is a vital process and has various effects on cell morphology, motility, proliferation and physiology [1], [2], [3]. The ability of adhesive cells to spread and migrate on a 2D or 3D substrate comes with the requirement to establish cell-matrix contacts that are stable enough to withstand traction forces but also dynamic enough to allow migration [4], [5], [6]. The connection between the intra- and extracellular domain is mediated by membrane-spanning integrins that directly connect to the extracellular ligands [7]. Within the large family of integrin receptors, a variety of ligands is found, such as fibronectin, vitronectin and collagen. Fibronectin is a dimeric protein composed of two identical 250 kDa strands connected via disulfide bonds at the C-terminus with each strand offering several motifs recognized as binding sites by the integrin family [8]. The shortest amino acid sequence known to be recognized as an adhesion motif is the RGD (Arginine-Glycine-Aspartic Acid) sequence located in FN repeat III_10_ serving as a binding site for α_5_β_1_, α_8_β_1,_ α_IIb_β_3_ and all α_v_ integrins [9], [10].

Integrin accumulation occurs in response to chemical and mechanical cues in their environment. Their interaction with the ECM leads to signaling cascades which eventually result in the accumulation of intracellular proteins into the cell-matrix contact sites resulting in the constitution and reinforcement of early adhesion sites [11]. Hereby a plaque of proteins is build at the adhesion sites and forms a direct link between the actin cytoskeleton and the ECM [12]. This link is vital to allow force transmission which is required for cell spreading and migration [13]. This is further exemplified by the finding that newly formed adhesion sides are not stabilized and disappear when the cell does not sense a sufficient counterforce provided by the environment [1], [13], [14], [15], [16], [17]. For integrin-mediated cell adhesion sites, it was shown that cells require a specific membrane/substrate interaction area to allow for the maturation of focal complexes into focal adhesions [13]. Furthermore, the distribution and density of integrin ligands on the substrate controls cell shape [2], [4], [18], proliferation rate, and adhesion forces [19], [20], [21], [22], [23].

Cell motility originates in the extension of the actin cytoskeleton by F-actin polymerization in the leading edge where migration is achieved by the exertion of inward facing traction forces [24]. During this process, transient early adhesions are reinforced into mature adhesion sites in response to force [1], [16], [25], [26]. In addition to the resulting forward cell movement, a retrograde flow of actin filaments is simultaneously observed which is initiated by forces exerted by myosin II that contract the cytoskeleton [27], [28], [29], [30], [31], [32]. The velocity of retrograde flow behaves reciprocal to the anterograde cell movement, leading to a fast retrograde flow in slowly migrating cells and vice versa [33], [34]. Concerning focal adhesion sites, a biphasic relation has been found between retrograde flow dynamics and traction stress [35].

The invention of optical tweezers in 1986 by A. Ashkin [36] added a versatile tool to established research techniques in the life sciences. Optical tweezers use strongly focused laser light to trap and move small dielectric particles and, due to their non-invasiveness, are nowadays widely used to study biological processes at the macromolecular scale [37], [38], [39], [40]. With optical tweezers high precision manipulation of objects on the cell surface or within the cell becomes feasible and in addition they can act as local probes to observe biological processes or biomolecular interactions [41], [42], [43], [44]. In combination with video microscopy, optical traps can give insight into force dynamics in the range of fN to pN with a temporal resolution of milliseconds. Therefore, optical tweezers have evolved into a versatile tool to study cell adhesion formation [1], [13], [19], [45], [46], [47], [48], [49], [50], [51], [52] and to measure cell force generation [39], [43], [53], [54], [55], [56], [57], [58], [59], [60] or membrane stiffness [61], [62], [63], [64]. With conventional single-trap optical tweezers, however, force development of only one individual adhesion site can be monitored.

To study the temporal development and strength of cellular traction forces at an extended number of individual adhesion sites, we established a custom-built multiple trap optical tweezers setup. The multiple trap feature of the optical tweezers system is a valuable tool to derive a force mapping across the entire cell surface. With the existing setup, we positioned up to 5 beads simultaneously on the apical membrane, which enables the coincidental initiation of adhesion sites in distinct locations. This allows the synchronous, time-resolved force mapping in adhesion sites with pN sensitivity. In addition, the influence of geometrical and spatial restrictions such as bead size and spatial relation of bead position was investigated.

As cellular forces and retrograde actin flow correlate [1], [28], [30], [35], [45], [46], [65], [66], [67], [68], we also used our system to study retrograde bead translocation in addition to force development at adhesion sites. Although several studies either demonstrate a link between traction forces and retrograde flow or a link between cell migration and retrograde flow [29], [31], [69], [70], to date no study correlating all three quantities has been undertaken. By applying identical sample preparation and imaging methods for three cell lines, we achieved a high degree of comparability, which enabled us to correlate the experimental results across different cell types.

## Results

### Multiple Trap Tweezers for Force Spectroscopy at Cell Adhesion Sites

**Figure 1:**
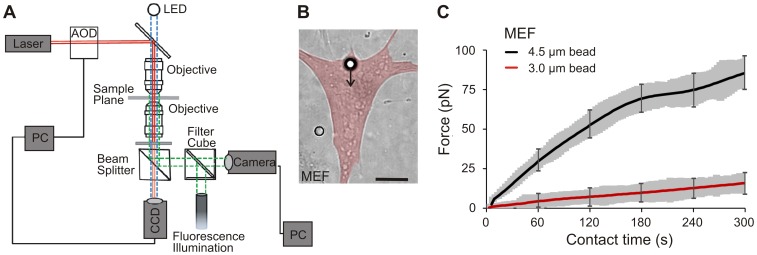
Experimental setup and cellular traction forces with regard to bead size. A) Schematic illustration of the multiple trap optical tweezers setup. The orthogonal alignment of two acousto-optic deflectors (AODs) allows for the simultaneous operation of several optical traps used to apply fibronectin functionalized beads to the cells. B) A fibronectin functionalized bead (3 µm diameter) is positioned by an optical trap in the leading edge of a cell. The arrow denotes the direction and magnitude of cellular traction forces. (a pseudocolored overlay was added to highlight the outline of the cell; scale bar = 5 µm). C) Beads with a diameter of 3 µm and 4.5 µm, respectively, were aligned on the cellular leading edge. The obtained averaged force-time curves only show reinforcement over the entire measurement intervall for 4.5 µm beads. No reinforcement occurs at cell contact sites established at 3 µm beads (mean ± s.e.m marked in gray and only exemplified for selected time points; N = 5, n = 14–17).

To investigate the cellular response induced by mechanical stimuli, we established a custom-built multiple trap optical tweezers system (Fig. 1 A) and provided it with live cell imaging equipment. Functionalized polystyrol beads were used to mimic new contact sites for cell-matrix adhesions and served as force probes to study the time-resolved development of force transduction. Before starting an experiment, the optical traps were calibrated in multiple trap mode. Two to eight beads were trapped simultaneously and the thermal fluctuations of the beads within the optical potential of the trap were recorded. By changing the AOD transmission, the laser intensity assigned to the traps was adjusted and thus the trap stiffness was controlled. The maximum laser power locatable to one individual optical trap was limited to 200 mW, which corresponded to a trap stiffness of 0.160 pN/nm for beads of 4.5 µm diameter and 0.100 pN/nm for beads of 3.0 µm diameter. Laser operation at 1064 nm offers low absorption rates in biological material, corresponding to a low risk for optical damage. Furthermore, the heating characteristics in aqueous solutions are suitable for live cell experiments [43], [71], [72]. By focusing the laser light onto polystyrol beads in an optical plane well above the cell surface, the level of irradiation of the cells is strongly reduced compared to the laser intensity in the trap center. In experiments with laser light focused for 30 minutes slightly above the apical cell surface, no change in cell morphology or proliferation was observed compared to control cells (data not shown). Experiments conducted in multi-trap mode allowed to expose cells simultaneously to a predefined number of beads to study the force development at distinct locations in one individual cell. Due to the fast scanning rate of the AOD system with up to 100 kHz, cells experience a quasi-static substrate rigidity upon the exertion of traction forces to the beads.

### Reinforcement of Adhesion Sites

FN functionalized beads were placed with optical traps on the apical cell surface of cells spread on a homogeneous FN-coated substrate to mimic new contact sites (Fig. 1 B). For the reinforcement of emerging adhesion sites, a counterforce to the cellular traction is required. Counterforces were applied to the beads by optical traps of fixed trap stiffness which could be chosen independently for each trap to be between 0.010 pN/nm and 0.160 pN/nm. During the formation of adhesion sites, cells apply traction forces to the beads, displacing them from the trap center. To study this process of force development in maturing adhesion sites, bead displacements were continuously analyzed over a time course of 5 min at a frame rate of 1 Hz. For a comparison of forces under different experimental conditions, the traction forces after an adhesion time of 5 minutes were regarded.

To determine the optimal conditions for the optical tweezers force spectroscopy assay, FN functionalized beads of 3.0 and 4.5 µm diameter were positioned by optical traps at the leading edge of mouse embryonic fibroblasts (MEFs) and the force development in the evolving adhesion sites was monitored (Fig. 1 C). Both bead batches were prepared with a FN surface coverage of 80% and each individual bead was confined to a trap with a spring constant of 0.100 pN/nm. For force measurements, a predefined number of beads were positioned onto the cell surface and optical traps were operated with a preset trap stiffness for 5 minutes. Cells form adhesion contacts around the beads as demonstrated by vinculin accumulation around the beads in transfected cells that express full length vinculin-GFP fusion proteins (Fig. S2). When the formation of adhesion sites was initiated, cellular traction forces were applied onto the beads. This resulted in a bead displacement from the trap center, which correlated linearly with the magnitude of exerted traction forces. Hence, the acquisition of bead displacement movies allows for a time-resolved analysis of cellular traction force development in early adhesion sites.

We obtained strongly reduced forces for 3.0 µm beads compared to forces transmitted onto 4.5 µm beads (F_3.0 µm_ = 16±1 pN after 5 min, number of independent experiments N = 5, number of examined cells n = 17, F_4.5 µm_ = 83±4 pN, N = 5, n = 14, Fig. 1 C). The contact area between microscopic beads and the cell membrane was calculated from the indentation depth of the bead, which was previously determined by atomic force microscopy and scanning electron microscopy to typically yield 0.05 to 0.2 µm [13], [73], [74]. From this, contact areas of ≤1.5 µm^2^ for 3 µm beads and ≤3.0 µm^2^ for the 4.5 µm beads were derived. In conclusion these experiments show, that a 1.5 times larger contact area induced a 5-fold increase in force transmission.

For all following force studies, functionalized 4.5 µm beads were used to mimic new contact sites as they geometrically enable maturation of initial adhesions into larger focal adhesions.

### Influence of Ligand Density on Force Development

The amount and spacing of substrate-bound integrin ligands in the extracellular domain has been shown to influence cell adhesion, proliferation, and migration [19], [21], [22], [75]. To determine the effect of ligand density on the investigated cell types, beads were functionalized with the ECM protein FN, a ligand offering adhesion motifs for a large number of different integrins. The shortest amino acid sequence known to be recognized as adhesion motif is the RGD sequence located in fibronectin domain III. We compared the influence of FN and cyclic RGD (cRGDfk) density on force development during the early assembly of cellular contact sites [75].

Beads were functionalized with different amounts of FN and cRGDfk, respectively, and batches with bead coverages of 50%, 80%, and 100% were prepared, with 100% corresponding to a completed ligand monolayer on the bead surface. The ligand density on the bead surface was controlled spectrophotometrically by optical density measurements. With optical traps the beads were placed on the leading edge of MEF cells and the displacement of beads from the trap center was monitored. Beads were restrained by traps set to the maximum trap stiffness of 0.160 pN/nm to provide the highest available resistance to the cellular traction forces and thus prevent the removal of the bead from the optical trap during the course of measurement.

**Figure 2:**
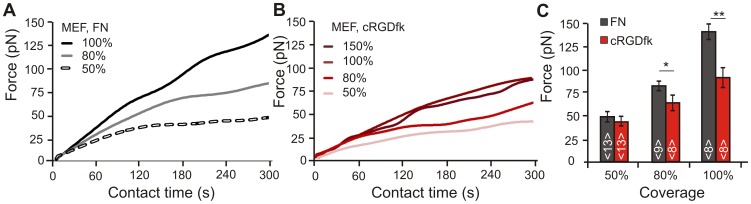
Dependence of cellular traction forces on ligand density. A) and B) Averaged force-time curves of cellular traction development with different coating densities of fibronectin (FN) or cRGDfk on 4.5 µm beads (averaged force-time curves including the s.e.m. for each considered ligand density are shown in Fig. S3) Cellular forces increase proportionally to the amount of ligand. C) Traction forces after 5 minutes observation time for FN or cRGDfk beads. Force exertion to FN beads is consistently higher as compared to cRGD beads (N = 5 for FN and for cRGDfk beads, n = 8–13 cells).

For MEF cells in contact with FN beads a dependence of the force-time curves on coating density was observed for membrane/bead interaction times longer than 60s (Fig. 2A). The evaluation of the force-time curves after 5 min yielded a correlation between coating density and force transmission (Fig. 2 C). This is supposedly due to enhanced integrin clustering mediated by the availability of high ligand densities. The force development on beads with distinct FN densities was distinguishable after less than 3 min of contact time (p<0.001). After 5 min of membrane/bead contact, cells transmitted forces of F_FN,50%_ = 49±3 pN (N = 5, n = 13), F_FN,80% = _81±5 pN (N = 5, n = 9) and F_FN,100%_ = 134±13 pN (N = 5, n = 8) onto the beads. Further enhanced coating densities were not tested with FN as ligand, as the forces applied to 100% FN beads were close to the maximum optical trap counterforces available with the existing setup.

Identical measurements were performed with beads functionalized with different densities of cRGDfk. Here, an additional bead batch with 150% surface coverage was prepared to test whether a saturation effect occurred. These beads had more ligands attached than required to form a complete monolayer and thus a second peptide layer formed. Data obtained with cRGDfk peptides show a dependence of both, the rate of force increase and the magnitude of forces on the coating density (Fig. 2B). The evaluation of the force-time curves after 5 min of membrane/bead contact shows a correlation between ligand density and force development for up to 100% bead coverage (Fig. 2 C). The forces obtained ranged from F_cRGD,50%_ = 44±7 pN (N = 5, n = 13) to F_cRGD,80%_ = 63±9 pN (N = 5, n = 8) and F_cRGD,100%_ = 92±11 pN (n = 8) and were discernible after 2 min of membrane-bead contact (p<0.0001). The formation of a second ligand layer on the bead (150% coverage) did not lead to an increased force transmission at the contact sites but resulted in a saturation on the force level observed for 100% coverage (F_cRGD,150%_ = 90±16 pN (n = 8)).

Control measurements with beads coated solely with PLL (without FN or cRGD) revealed an unspecific bead attachment to the cell surface of a small percentage of beads (about 8%). Although these beads were in contact with the membrane, they did not show rearward translocation and could easily be displaced by the optical traps.

Thus, traction forces in the initial 5 min of cell adhesion formation depend on the density of integrins ligands offered on the bead surface. A comparison of cellular traction forces applied to beads covered with different densities of FN and cRGD displays enhanced force transmission at contact sites formed to FN functionalized beads (Fig. 2C). This divergence of force transmission on different ligands becomes more pronounced with enhanced ligand densities.

### Influence of Bead Position on Force Development

**Figure 3:**
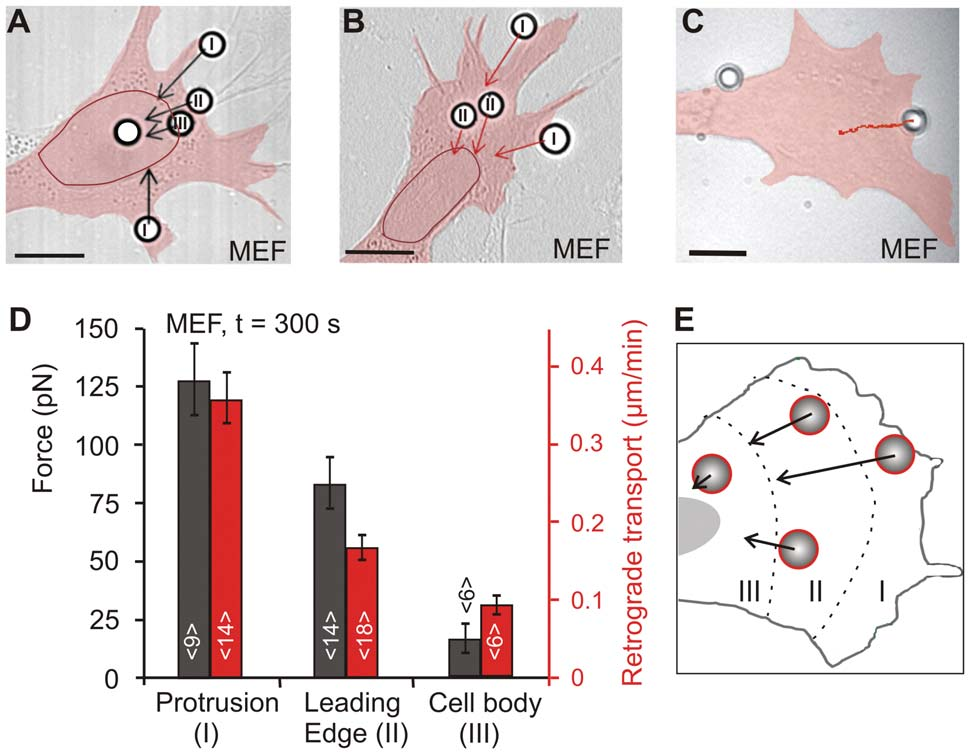
Mapping of traction force and velocity onto the cell surface. A) and B) DIC images of FN-beads deposited at various positions onto the surface of MEF cells. The cell surface is classified into three areas with area I referring to cellular protrusion, area II to the leading edge and area III to the cell body. A) For force measurements beads were positioned onto the cell surface and optical traps were operated with a predefined trap stiffness of 0.160 pN/nm for a time course of 5 minutes. Black arrows denote the direction and magnitude of cellular traction forces. B) For measurements of retrograde transport velocities, beads were positioned by optical traps onto the cell surface, traps were switched off after 5 seconds, and bead movements were recorded in a time lapse movie. Red arrows denote the direction and velocity of retrograde transport. C) Example trajectory of an individual bead positioned in area I (a pseudocolored overlay was added to highlight the outline of the cells and the nucleus was traced by a red line; scale bar = 10 µm). D and E) Forces and retrograde transport velocities are position-dependent and correlate with each other (N = 5).

The optical tweezers setup features a multi trap mode allowing to measure traction forces simultaneously at distinct locations. This enables a force mapping with high spatial and temporal resolution over the whole cell body of a single cell and grants a high accuracy when comparing forces measured within distinct cellular regions. Here, we simultaneously arranged a predefined number of beads on cellular protrusions (area I), the leading edge (area II) and the cell body (area III) of a single cell to estimate the dynamics and adhesion strength occurring during initial force development (Fig. 3 A to D). All beads were functionalized with a FN surface coverage of 80% and were captured in traps featuring identical spring constants in multimode operation.

Traction forces were highest in the foremost protrusions, decreased rearwards toward the leading edge and became negligible when beads were placed in the nuclear region or the rear of the cell (Fig. 3 D and E). The example in figure 3 A depicts a cell with 5 beads distributed across the surface: two beads were positioned in area I, one at a forward directed protrusion and one at a lateral directed protrusion, another bead was located in area II and one bead was placed in area III. One bead was attached to the rather static nuclear area and was not considered for force evaluation as the formation of new adhesion sites is not expected in this area. The force vectors attributed to the bead in area I correspond to the highest forces in this area relative to areas II and III. Beads located on the leading edge experienced cellular traction forces in an intermediate force regime while hardly any force development was observed for beads placed in area III. Furthermore, the force mapping approach demonstrated that adhesion sites exhibit similar traction forces when they are located at an equal distance from the foremost tip.

We conducted complementary measurements to evaluate the rate of retrograde bead transport in different cellular areas (Fig. 3 B and C). Functionalized 4.5 µm beads with a FN coverage of 80% were positioned with optical traps on the cell membrane and traps were switched of instantly to monitor the rearward bead translocation. As the beads were large compared to topographical changes in the cell surface, they stayed in the focal plane and were easily traceable. The retrograde transport velocity of FN functionalized beads was fastest at protrusions and decreased steadily when the distance to the membrane tip was enlarged (Fig. 3 D). This is consistent with the results obtained for cellular traction forces and reveals an intrinsic relation of force generation and retrograde flow rates.

### Geometrical Constraints Limit Force Transmission

**Figure 4:**
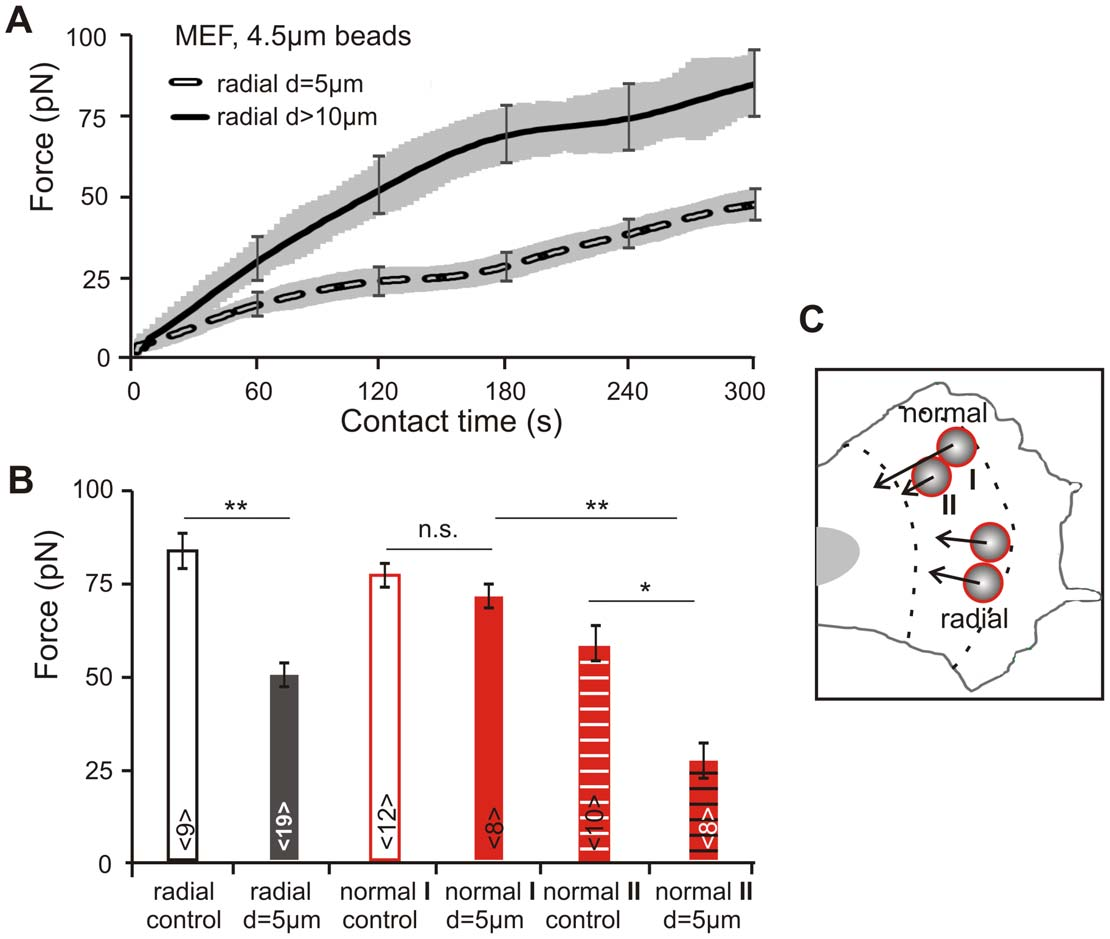
Influence of bead distance on force development. A) Pairs of 4.5 µm beads were positioned in a radial orientation with a center-to-center spacing of 5 or 10 µm. The averaged force-time curves show a force development similar to single beads for distances of more than 10 µm between adjacent beads (mean ± s.e.m marked in gray and only exemplified for selected time points). With bead spacing of 5 µm, reinforcement of each neighboring adhesion site was reduced to 60%. B and C) Two bead orientations with bead spacing of 5 or 10 µm were considered: radial and normal with respect to the leading edge, respectively. Cellular traction forces applied to the normal oriented anterior bead I were significantly enhanced compared to forces acting on the posterior bead II. Control measurements were performed separately for the locations of bead I and II: in control I the anterior bead was positioned in an identical distance to the tip of leading edge as bead I, and the second bead was displaced by 10 µm toward the nucleus. For control II, the posterior bead was placed in the same distance to the leading edge tip as bead II, and the paired bead was displaced by 10 µm toward the leading edge. This procedure was necessary, as traction forces at adhesion sites decrease with increasing distance to the leading edge (N = 5, n = 8–19 cells).

In the cell periphery, cell matrix adhesion sites are often spatially closely related. The results presented so far all considered adhesion sites that were well separated. In the force mapping assay, multiple beads on the cell surface were spatially separated by distances >10 µm and therefore were regarded as independent. Now, we tested the influence of spatially closely related beads and adhesions on force transmission. The beads offering new contact sites for the cells were placed with a distance of 5 µm on the leading edge of the cell (4.5 µm beads, 80% FN surface coverage, 0.080 pN/nm trap stiffness). A control was performed with bead spacing larger than 10 µm and yielded the same result as obtained for force transmission on single beads. Two examples of bead spacing were regarded for the acquisition of force-time curves: (i) beads were placed in a radial orientation and (ii) beads were placed in successive order and were aligned normally to the border of the leading edge (Fig. 4 C).

In case (i), a declined force transmission was observed when the spatial separation between beads was reduced from 10 to 5 µm (Fig. 4 A to C). On 5 µm spaced beads, cells applied traction forces amounting about 60% of the strength they exerted on spatially separated beads (F_5 µm = _49±4 pN; N = 7, n = 19) compared to F_10 µm = _83±4 pN (N = 5, n = 9). The force-time curves show that the force development becomes divergent already after 30 seconds of membrane/bead contact time (p = 0.02).

The second case (ii) with a normal bead orientation (Fig. 4 B and C) yielded a stronger force reduction than case (i) when the distance between the beads was reduced to 5 µm. Here, the bead closer to the leading edge (bead I) does not experience any reduction in force transduction but shows the same behavior as a single bead. Instead, reduced force transmission occurred on bead II where cells created only 38% of the forces applied to the anterior bead (F_5 µm = _70±4 pN; N = 5, n = 8 and F_10 µm_ = 26±5 pN; N = 4, n = 7).

### Relation between Force, Retrograde Transport, and Cell Migration in Different Cell Types

In a number of studies, cellular traction forces have been examined using multitudinous approaches [16], [61], [76], [77], [78], [79], [80], [81]. The measured forces varied strongly depending on the cell type used. However, they were not easily comparable due to the diversity of applied methods. Here, we study three different cell types, namely B16 cells, MEFs and PCFs, with the same optical setup, identical sample preparation, and identical imaging conditions to allow for a comparison of force development. In addition to traction forces we measured the retrograde transport velocity and cell migration rate for the three cell types. Again, 4.5 µm beads were functionalized with FN to a surface coverage of 80% and were restrained by traps with a spring constant of 0.160 pN/nm. The intermediate surface coverage was chosen to ensure an optimal utilization of the full force range accessible by the optical tweezers setup (10 to 160 pN).

**Figure 5:**
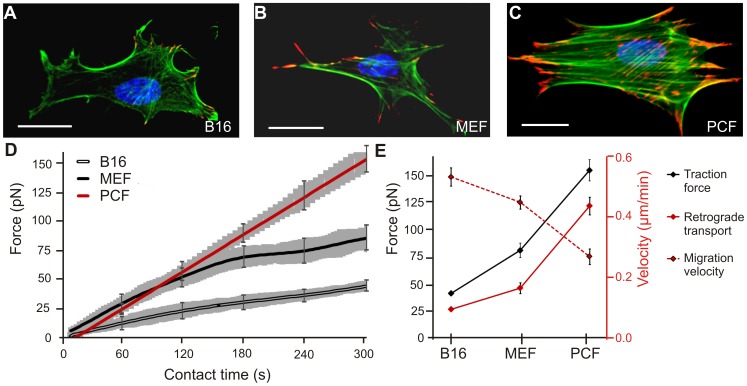
Comparison of different cell types. A) to C) Fluorescent labeling of the nucleus (blue) actin cytoskeleton (green), and vinculin (red) of mouse B16F1 melanoma cells (B16), mouse embryonic fibroblasts (MEF) and primary chicken fibroblasts (PCF) (scale bars 20 µm). D) Averaged force-time curves for the three cell lines (s.e.m. is marked in gray and only exemplified for selected time points) using 4.5 µm beads with 80% FN surface coverage. E) Correlation of traction forces, retrograde transport velocities, and migration velocities for the different cell types. A linear correlation for force and retrograde transport is revealed and is concomitant with a reciprocal correlation for migration velocity.

The immortalized B16 and MEF cells were of similar size and featured an elongated shape (Fig. S1). In contrast, the PCF cells were much larger and adopted a more squared morphology. The analysis of the structural organization of the actin cytoskeleton showed that B16 melanoma cells developed a random meshwork of actin fibers in the leading edge, with characteristic arc-like fibrils connecting neighboring peripheral adhesion sites (Fig. 5 A). MEF cells displayed more pronounced actin stress fibers originating from the adhesion sites in the cell periphery and stretching towards the cell center (Fig. 5 B). The adhesion sites in both cell lines (marked by staining of endogenous vinculin) were of similar shape and size. Distinctively, PCF cells showed large clusters of vinculin containing adhesion sites in the leading edge but displayed smaller individual adhesions in the central area (Fig. 5 C). Furthermore, PCF cells featured an extensive actin stress fiber system spanning the length of the entire cell.

Traction forces were measured at the contact site of a 4.5 µm FN functionalized bead over a time course of 300 s. The forces transmitted during the formation of adhesive contacts were clearly distinguishable for the three investigated cell types and after 300 s the following forces resulted (Fig. 5 D): F_B16_ = 42±2 pN (N = 5, n = 12), F_MEF = _83±4 pN (N = 6, n = 14), F_PCF = _151±10 pN (N = 7, n = 20). B16 cells showed the slowest increase of traction forces over time and after 1 min a significant difference in force transmission was observed in comparison to MEF and PCF cells (p<0.01).

MEF traction forces evolve in a two-step process with a steep force increase in the early adhesion formation (initial 120 to 180 s) followed by a moderate force development in the second phase. Concerning the entire course of measurements, we derived the fast reinforcement rate for PCF cells. Although PCF and MEF cells showed the same traction force evolution in the first 120 s of bead contact, MEF force enhancement slowed in the following, while PCF cells maintained a linear increase in adhesion strengthening and exerted steadily growing forces onto the beads. The measurement duration was extended to 10 min to determine whether PCF reinforcement continued at the same rate: no decrease in adhesion strengthening was found within the increased measurement interval and after 8 min the cells overcame the optical counterforce and removed the beads from the traps (data not shown).

In addition to traction forces, retrograde transport dynamics were characterized for B16, MEF and PCF cells. Since beads with 100% FN coverage are detached from the traps in less than 5 minutes by PCF cells, 4.5 µm beads with 80% FN surface coverage were placed on the leading edge of the cells. Lacking a restoring force from the optical traps, beads attached to the cell surface were coupled to the retrograde actin flow and move rearward towards the nucleus. Bead trajectories were derived from time lapse records with a frame rate of 1 Hz over a time course of 20 min. The evaluation of the retrograde transport velocity v_rt_ resulted in the fastest transport dynamics in the PCF cells, followed by moderate transport rates for MEF cells and slow transport dynamics for B16 cells (v_rt,PCF = _0.44±0.03 µm/min, N = 5, n = 21, v_rt,MEF = _0.17±0.02 µm/min, N = 5, n = 24, v_rt,B16_ = 0.097±0.006 µm/min, N = 6, n = 36). A cell type comparative analysis of force transmission and retrograde transport shows a linear correlation of the two values (Fig. 5 E), and reveals an intrinsic link of force generation and actin flow dynamics.

The retrograde transport velocity is known to inversely correlate with cell migration velocity [27], [33], [82]. We tested this relation with the three cell lines chosen for force and retrograde transport studies. Time lapse images were recorded with 1 frame per min over a time course of 12 h and the migration velocity v_m_ was derived by tracking the movement of the nucleus. The fastest migration speed was found in B16 cells, followed by MEF cells and PCFs: v_m,B16_ = 0.53±0.03 µm/min, N = 5, n = 53; v_m,MEF_ = 0.44±0.02 µm/min, n = 49; v_m,PCF = _0.27±0.03 µm/min, N = 5, n = 51 with p<0.02 (Fig. 5 E).

Comparing the results for traction forces, retrograde transport rates, and migration velocities in B16, MEF and PCF cells, a linear relation between force and retrograde transport velocity is apparent: a high retrograde transport velocity is associated to strong cellular traction forces and vice versa. In addition, a reciprocal behavior is observed between cell migration velocity compared to traction force and retrograde transport rate. This corresponds to fast migrating cells exerting the lowest traction forces at developing adhesion sites.

## Discussion

Although the process of force generation has been studied with various approaches, many details about the interaction of the intracellular cytoskeleton with the extracellular surrounding remain elusive. The multiple trap optical tweezers setup allows for force spectroscopy with local force probes. Functionalized beads act as passive sensors for cellular traction forces and do not actively apply forces to the cell. Hence, the cellular response to ligand stimuli is not subjected to external mechanical disturbances, which enables a noninvasive study of evolving adhesion sites. By changing the optical trap intensity, the compliance of the force probe can be adapted to mimic specific substrate rigidities. The resistance of trap confined beads to cellular traction forces is much lower than the compliance of the commonly used glass substrates and reflects the physiological conditions of cell-matrix interactions more closely. It should be noted, however, that traction forces in our experimental setup are measured on the dorsal side of adherent cells. It is known that the organization of the actin cytoskeleton differs between the ventral and dorsal side and also depends on adhesion conditions [83].

Crucial parameters for cellular traction force generation are the size of the adhesion area, the ligand density, adhesion site position with regard to the leading edge, and the spatial separation of adjacent adhesion sites. We evaluated these parameters to quantify and characterize the development of forces in individual adhesion sites immediately after adhesion initiation.

The formation and development of adhesion sites was analyzed with regard to the available contact area between cell surface and bead. The dot-like initial adhesion sites reach a size of 1 µm diameter and mature into elliptical focal adhesions with an expansion of 2 to 5 µm along the elongated axis. It has been shown that the formation of mature adhesion sites requires a sufficient contact area between bead and cell, with bead diameters of about 3 µm matching this criterion [13]. Dependence between adhesion size and force development has also been shown in cells cultured on flexible substrates [84]. Our analysis of adhesion forces in MEF cells showed that 4.5 µm beads were best suited for studies of adhesion site reinforcement as the membrane/bead contact area allowed the maturation of initial adhesion sites into mature adhesion sites. In contrast, the membrane/bead contact area provided by 3.0 µm beads did not suffice to induce adhesion reinforcement. The reduction of the available contact area by a factor of two resulted in a 5-fold decrease of the cellular traction forces, although the ligand density on the bead surface was kept constant. This nonlinear relation between contact area and traction force indicates a size dependent change in the protein constitution of the adhesion sites and shows that in addition to the counterforce a sufficiently large contact area has to be available to transmit high forces [13].

Apart from geometrical restrictions, the amount of integrin ligands available in the extracellular environment is a limiting factor to adhesion formation and reinforcement. The type of ligand [9], [19], ligand density [3], [21], [75], [85] and ligand spacing [2], [4], [23], [86] regulate adhesion formation and force transmission. To examine the ligand affinity of cells, force transmission on beads functionalized with the ECM constituent FN were compared to cRGDfk coated beads. FN is a macromolecule comprising distinctive adhesion motifs, which serve as binding sites for a large number of different types of integrins. Among others, the tripeptide RGD sequence in the FNIII_7–10_ domain mediates the formation of cell-substrate adhesions. The RGD sequence is known to be recognized by several integrins and is arranged in a loop-like conformation in the wild type FN domain III. The cyclic tripeptide cRGDfk was chosen for the experiments as the cyclic conformation is supposed to resemble the wild type situation more closely than a linear peptide configuration [87]. We administered ligand functionalized beads with a surface coverage ranging from 50% to 100%, where 100% surface coverage corresponded to a tightly packed ligand monolayer. The force transmission to FN- and cRGDfk-beads in contact with MEF cells revealed an adhesion reinforcement that was corresponding to the increase in ligand density. To determine whether adhesion reinforcement continued for ligand amounts exceeding the amount required to form a ligand monolayer, we prepared a bead batch with a cRGDfk coverage of 150%. This relates to the formation of a ligand bilayer on the bead surface. We observed cellular traction forces of the same magnitude as for beads coated in a ligand monolayer. The monitored saturation effect could be caused by impaired binding properties in the upper ligand layer, were peptides are bound to the underlying peptide layer only. cRGDfk-cRGDfk interaction is considered to be less stable and less resistant to cellular traction force application and thus might facilitate peptide detachment from the bead surface. Furthermore, the phenomenon could be attributed to an excess of ligand molecules, which cannot be assessed by integrins. Due to spatial restrictions within the adhesion site these ligands cannot contribute to enhance integrin clustering, resulting in a saturation of force transmission at the contact site.

The comparative study of the two ligands FN and cRGDfk gave evidence to enhanced force transmission at adhesion sites mediated by FN-beads. The preference for FN comprising substrates could be attributed to the capacity of FN to bind to a wider range of integrin subunits. For example, the integrin α_5_β_1_ binds to FN, but not to the RGD adhesion motif alone [19]. Instead, α_v_β_3_ integrins promote RGD binding. α_5_β_1_ is a key regulator of adhesion reinforcement and resists high traction forces, while α_v_β_3_ is less stable under high tension but mediates signal transduction [88]. Hence, the observed reinforcement gain of FN-integrin complexes over cRGDfk-integrin adhesions might be induced by the role of distinctive integrin subunits in adhesion regulation.

With the multiple trap feature of the optical tweezers setup, it is feasible to study the cellular traction forces exerted onto ligand-coated beads positioned in distinct areas of the cell membrane. Furthermore, beads functionalized with the ECM proteins can be used to study cytoskeletal dynamics and were among the earliest approaches to study retrograde actin flow in fibroblasts [1], [45]. We performed complementary measurements on traction force development and retrograde transport velocities in distinct cellular areas and found an intrinsic correlation of the two parameters [35], [68], [77], [89]. Both, forces and velocities were highest in the foremost protrusions of MEF cells and decreased with increasing distance to the foremost membrane tip [5], [90].

Cellular traction forces depend on multiple parameters, such as ligand density, surface rigidity, and the size of adhesions. Here, we tested whether the spatial separation of individual adhesion sites influences their evolution and force transmission. To probe their environment cells form membrane extensions in the lamellipodium. In this highly dynamic area, a large number of adhesion complexes are constantly assembled and remodeled. Due to this, tightly arranged adhesion patterns move towards the lamella where they mature into focal adhesions or disassemble. Hence, the evolution of adhesion forces in neighboring adhesion sites and the spatiotemporal coordination of adhesion reinforcement are of particular interest. Adhesion sites that developed with a spatial separation of 10 µm or more displayed the same behavior as isolated adhesion sites. However, the reduction of the center-to-center distance of neighboring adhesions to 5 µm led to significant changes in force transmission. When two beads were radially aligned on the leading edge, both beads experienced the same force magnitude and these forces were reduced by about 40% compared to independent adhesion sites. The force reduction could be created by the competition of the two emerging adhesion sites for adhesion-mediating proteins from the cytoplasmic pool. In addition, the closely neighboring adhesions could be linked to a set of underlying actin fibrils which is supported by a single actin stress fiber. This could also explain the apparent synchronization of the force development in the two adhesion sites. However, further investigations are required to analyze the reorientation of the actin network and possible mechanisms for adhesion crosstalk.

We also studied the effect of the intrinsic orientation of a set of closely related adhesion sites. Therefore, we arranged two beads normally and evaluated the forces transmitted to the forward and successive bead. Our data showed a decrease of adhesion forces on the successive bead. The decline of traction forces exceeded the expected reduction due to an enlarged distance to the leading edge, which was confirmed by control measurements at independent adhesions [17], [70], [91], [92], [93], [94], [95]. It has been proposed that adhesion sites constitute a barrier for retrograde actin flow by inducing friction and thus decelerate the actin flow [32], [33], [70], [96], [97]. A theoretical model has been developed that predicts a stretching of the rearward flowing actin network upon encounter with the adhesion sites, which results in a stress-dependent partial disintegration [90], [98], [99], [100], [101], [102]. The model computed by Shemesh and coworkers projects the appearance of “shadows” of low actin density right behind adhesion sites. Hence, the reduced forces in an adhesion site closely succeeding an anterior adhesion could be induced by friction between the cytoskeleton and adhesion sites. As the rearward bead is situated in the shadow region of its predecessor it lacks access to the actin network and is impaired in reinforcement. Together, this indicates that reinforcement of adhesion sites does not only depend on the parameters of the contact directly concerned but also relies on neighboring adhesions.

Our data reveal that both traction forces and retrograde flow velocity decrease with distance to the leading edge. This result agrees well with an unperturbed flow of the underlying actin cytoskeleton initiated by polymerization at the membrane tip. By contrast, the work of Gardel and coworkers on ventral adhesions showed a biphasic relation of force and velocity: initially, a decrease of flow velocity was accompanied by an increase of forces [35]. The organization of the actin cytoskeleton differs between the ventral and dorsal side [83]. Hence, a relation between the maturation of focal complexes into focal adhesions to the spatial organization of the cytoskeleton at the transition from lamellipodia to lamella should to be considered [103].

In addition to the analysis of cellular traction forces and retrograde flow velocities on the scale of individual MEF cells, we performed a related cell type comparative study. Furthermore, we took the cell migration velocity into consideration. For the three cell lines (B16, MEF, PCF) investigated, our data reveal a cell type independent correlation of traction force generation at adhesion sites, retrograde transport rates and cell migration velocity. B16 melanoma cells developed the weakest forces, which correlated to slow retrograde transport rates. Concomitantly, these cells featured the highest migration velocities of all three cell lines. MEF cells displayed forces, retrograde transport velocities and migration rates in an intermediate regime, while the primary cell line of PCFs exhibited the strongest forces, fastest retrograde transport and slowest migration.

A correlation of traction forces and retrograde actin flow has been demonstrated in previous studies [35], [68], [77], [104] as well as an inverse relation of retrograde flow and cell motility [31], [105]. However, traction forces, retrograde flow velocities and migration rates together have not yet been analyzed in a cell type comparative manner. Our data confirm the conservation of the proportionality of forces and actin flow velocity as well as the inverse correlation of forces and actin flow with migration velocities throughout different cell lines.

## Materials and Methods

### Ethics Statement

Fertilized chick eggs (Gallus gallus domesticus) used for the preparation of primary chick fibroblasts were obtained from a local breeder and experiments were performed according to European (Council Directive 86/609/EEC) and German (Tierschutzgesetz) guidelines for the welfare of experimental animals. Embryos were dissected from eggs after 8 days of breeding in a commercial egg incubator and sacrificed by decapitation prior to skin preparation.

### Cell Culture

Mouse B16F1 melanoma cells (B16 cells; kindly provided by B. Imhof, CMU- Universite de Geneve, Switzerland) [106], mouse embryonic fibroblasts (MEF cells; kindly provided by W.Ziegler, IZKF Leipzig, Germany) [107] and primary chicken fibroblasts (PCF; isolated from the skin of day 8 chicken embryos) were cultured at 37°C under humidified atmosphere and 5% CO_2_. B16 and MEF cells were maintained in Dulbecco’s modified Eagle’s medium (DMEM, Invitrogen) supplemented with 10% fetal bovine serum (FBS, Sigma-Aldrich) and PCFs were supplied with Kaighn’s modified nutrient mixture (Invitrogen) with 10% FBS and 5% chicken serum. Prior to experiments, cells were plated onto fibronectin (FN) functionalized glass-bottom culture dishes (MatTek) and preincubated for 2 hours to allow cells to spread. 30 min before measurements the standard growth medium was exchanged for CO_2_ independent medium (phenol-red-free DMEM or F-12 containing 20 mM Hepes and 2% FBS and during the course of measurements cells were kept in a live cell imaging chamber at 37°C.

### Fluorescent Staining

Monoclonal mouse anti-vinculin antibodies (clone hVIN-1, Sigma Aldrich) were used in combination with Cy3-conjugated goat-anti mouse F(ab’)2 antibodies (Dianova) to mark focal adhesions. The actin cytoskeleton was stained with Alexa488-conjugated phalloidin (Invitrogen) and the nucleus was visualized with DAPI (Sigma Aldrich).

### Vinculin Expression

MEF cells were transfected to express full length vinculin-GFP fusion proteins (pEGFP-C2 vectors, Clonetech). 2 µg plasmid DNA were transferred into an electroporation cuvette and were mixed with 10^6^ cells suspended in 250 µl cold electroporation buffer (120 mM KCl, 10 mM K_2_PO_4_/KH_2_PO_4_, 2 mM MgCl_2_, 25 mM Hepes, 0.5% Ficoll 400; pH7.6). The cuvette was incubated on ice for 2 min before subjection to an electroporation pulse of 250 V and 60 ms duration (Gene Pulser Xcell, Bio-Rad). Subsequently the cell suspension was stored on ice for 2 min and cells were mixed with DMEM medium and replated onto cover slips. The transfected cells were incubated for 16 h under routine conditions to express the protein. Subsequently, cell samples were incubated with FN-functionalized beads for 20 min and were fixed to analyze vinculin recruitment at bead contact with a confocal laser scanning microscope (LSM 510, Carl Zeiss).

### Bead Functionalization

Carboxylated polystyrene beads of 3.0 µm and 4.5 µm diameter (Polysciences) were homogeneously functionalized either with the extracellular matrix protein FN (human plasma fibronectin, 1 mg/ml; Sigma-Aldrich) or with a cyclic RGD peptide (c(RGDfk)-(Ahx)_3_-N_3_, 1 mg/ml). Therefore, beads were incubated with 100 µl Poly-L-Lysine in PBS (PLL, 200 µg/ml, Sigma-Aldrich) for 1 h at room temperature with gentle mixing. The solution was washed 3 times with PBS by centrifugation and varying amounts of FN (1 µl, 2 µl, 3 µl) or cRGDfk (2 µl, 4 µl, 5 µl, 8 µl) were added into 100 µl PBS to obtain distinct densities on the bead surface. Beads were incubated with gentle mixing for another hour at room temperature. The amount of ligand attached to the bead surface was determined spectrophotometrically by UV-VIS spectroscopy (NanoDrop1000, PEQLAB Biotech-nologie GmbH). Optical density data were obtained from the supernatant of the bead-ligand solution at a wavelength of 260 nm and yielded the concentration c_residue_ of residual (unattached) ligand. The number of ligand molecules in the supernatant was calculated from the relation N_residue_ = c_residue_ VN_A_ (V denotes the volume of the solution and N_A_ the Avogadro constant). From the number of initially deployed ligand N_ini_ and residual ligand N_residual_, the number of attached ligand molecules per bead N_b_ was estimated: N_b_ = (N_ini_ – N_residue_)/n_b_ where n_b_ is the number of beads. The size of an individual 450 kDa FN molecule was determined by scanning electron microscopy [108], [109] and amounts a width of 2 to 3 nm and a length of 61 nm. Thus, an individual FN molecule can cover an area of approximately 120 nm^2^. For the 1 kDa cRGD tripeptide, a surface coverage of about 0.24 nm^2^ per molecule was assumed from the relation of FN to cRGD molecular weights. From this calculation, the degree of surface coverage can be estimated. For the presented experiments, bead batches with a surface coverage of 50%, 80% and 100% were prepared for the ligands FN and cRGDfk. An additional sample with 150% coverage was prepared with the cyclic peptide only. Bead-ligand solutions were washed 3 times with PBS to remove residual ligands and were finally stored in 1× PBS at 4°C. Beads were freshly prepared one day prior to the experiments.

### Substrate Preparation

Glass bottoms of culture dishes (MatTek) were covered with a solution of 50 µl PLL in PBS and incubated for 1 h at room temperature. After rinsing the dishes twice with PBS, 0.5 µl FN diluted in 50 µl PBS were added and dishes were incubated for 1 h at room temperature. Residual FN was removed by rinsing twice with PBS and the culture dishes were stored at 4°C covered with PBS until further use.

### Multiple Trap Optical Tweezers

The laser tweezers setup was based on a two-axis acousto-optical deflection system (AOD, AAoptoelectonics) which allowed the independent steering and intensity modulation of a large number of quasi static optical traps. Optical traps were operated by an AOD beam steering controller (Aresis), allowing closed-loop operation by relying on the live image of a CCD camera (C3077, Hamamatsu Photonics) for positioning of optical traps. Due to the fast access time (6.5 µs) of the deflectors, multiple time-shared traps could be generated within a scan angle of 200×200 µm and operated simultaneously. An infrared diode pumped solid state laser (Compass 1064; Coherent), operating at a wavelength of 1064 nm, was coupled into a custom-built microscope and focused by a high numerical aperture objective to generate the optical traps. The microscope was equipped with epifluorescence optics and a heating unit to maintain a sample temperature of 37°C for live cell force measurements. To manipulate microscopic beads on the cells surface, a water immersion objective (63×, 1.2 numerical aperture, Carl Zeiss) was lowered into the imaging medium within of a glass bottom dish. Imaging was performed with an oil immersion objective (63×, 1.2 numerical aperture, Carl Zeiss) mounted below the sample dish.

### Optical Trap Calibration

The optical traps were calibrated using the thermal fluctuation method in combination with video microscopy for trap stiffness and the drag force method for maximum trap force determination [40].

Optical trap calibration was performed prior to the experiments in multi trap mode to ensure that no additional losses due to the scanning of the laser beam reduced the effective trap stiffness. Geometrical patterns of four to eight optical traps were generated and calibrated simultaneously (Fig. 1 B). Each trap was assigned to an individual laser intensity and a polystyrol bead of 3.0 µm or 4.5 µm was captured in the center of each trap. Thermal fluctuations were recorded for a time course of 10 min with the CCD camera and the resulting bead trajectories were obtained with MetaMorph (Visitron) and evaluated with MATLAB (MathWorks). From the distribution of bead displacements from the equilibrium position in the trap center, the optical potential *U(x)* of each trap was derived: U(x) = k*Δx(t)^2^ where *Δx* is the bead displacement and *k* the trap stiffness. The optical potential was calculated separately for forces pointing in x and y direction and showed a circular symmetry. Calibrations were performed close to the apical cell surface. The laser intensity applied to a trap was adjusted by changing the AOD transmission via the software interface Tweez (Aresis). The recorded video frames were submitted to data analysis to obtain the trap stiffness for each trap for varying laser power.

To determine the maximal optical force on microscopic beads, the drag force method was applied. A bead was captured in a trap well above the cell surface and moved through the sample plane with a defined trap velocity. The trap velocity was enhanced until the bead was unable to follow the trap. From the recorded escape velocity the maximum trap force was calculated using the Stokes relation *F = βv = 6πηrv*, where *β* is the drag coefficient, *v* is the trap velocity, *η* denotes the fluid viscosity and r the bead radius.

The linear dependence of trap force on bead displacement F(t) = k*Δx(t) is utilized to determine the traction forces a cell applies to a trapped bead.

### Force Spectroscopy Assay

Cells were transferred onto FN functionalized glass bottom dishes and were incubated for two to three hours to allow cells to spread. Half an hour before a measurement, the medium was exchanged for F12 imaging medium containing 20 mM Hepes and 2% FBS. FN functionalized beads were applied into the culture dish and 1 to 5 optical traps were activated to capture the beads and position them on the cell. Beads were placed on individual cells with positions varying between cellular protrusions, the leading edge and the cell body. Traps remained active during the complete measurement course of 5 min and images were recorded with a frame rate of 1 Hz with the CCD camera.

### Retrograde Transport Assay

To characterize the dynamics of retrograde actin flow, the retrograde transport of microscopic beads was monitored. Cells and beads were prepared as described and a predefined number of beads were positioned by optical traps on the cell surface. For the retrograde transport assay, optical traps were switched off immediately after positioning the beads. The bead position was recorded with the CCD camera over a time course of 20 min and with a frame rate of 0.5 Hz. The videos were analyzed with MetaMorph to determine the bead position in each frame and the retrograde transport velocity of the beads was calculated from the derived trajectory data.

### Cell Migration Assay

To characterize cell motility, cells were sparsely plated onto FN functionalized glass bottom dishes and preincubated for 2 h at 37°C and 5% CO_2_ to allow for adhesion and spreading. Subsequently, time lapse movies of migrating cells were recorded (Colibri/AxioObserver.Z1, 20×/0.8 Ph2 plan- apochromate, Carl Zeiss) with a rate of 1 frame per minute for 12 hours.

### Image Analysis

To investigate force development and retrograde transport, time lapse movies were recorded at a frame rate of 1 Hz and analyzed with the object tracking application of MetaMorph (Molecular Devices). In each frame the actual bead position was determined by the software and the bead displacement was calculated as the distance between trap center and actual position.

The velocity of migrating cells was derived from the observation of the locomotion of its nucleus. The nucleus was tracked with the manual tracking plugin (F.Cordelieres, NIH ImageJ) and the nuclear velocity was calculated with the chemotaxis and migration plugin (ibidi) for ImageJ.

## Supporting Information

Figure S1 **DIC images of the three cell types studied.** Cells were seeded onto homogeneously fibronectin-functionalized glass cover slips and incubated for 2 hours before fixation. A) Mouse melanoma B16F1 cells, B) mouse embryonic fibroblast (MEF) cells and C) primary chick fibroblast (PCF) cells (scale bars = 100 µm).
(TIF)

Figure S2 **To test whether cells accepted the FN functionalized beads to invoke new adhesion sites on the surface, MEF cells were transfected to express a full length vinculin-GFP fusion protein.** In figure S2 A) the accumulation of vinculin at the membrane/bead interface is depicted. The bead on the cellular leading edge did not experience any external force as it was not restrained by the optical forces of the laser trap. In about 50% of the examined cells a vinculin-GFP circle had formed around the bead, confirming the formation of adhesion sites at the contact area. B) Overlay of a DIC image with the fluorescent channel (scale bar = 10 µm).
(TIF)

Figure S3 **Averaged force-time curves for each surface density of FN and cRGDfk functionalized beads.** A) to C) show the development of cellular traction force exertion onto FN-beads and D) to G) onto cRGDfk-beads (mean ± s.e.m; the s.e.m. is denoted in gray and the gray bars are error bars representing specific time points, N = 5, n = 8–13).
(TIF)
