# Supplementary figures and images for: Force Mapping during the Formation and Maturation of Cell Adhesion Sites with Multiple Optical Tweezers
Source: PLoS One. 2013 Jan 25;8(1):e54850. doi: 10.1371/journal.pone.0054850 (PMC3556026; doi:10.1371/journal.pone.0054850)

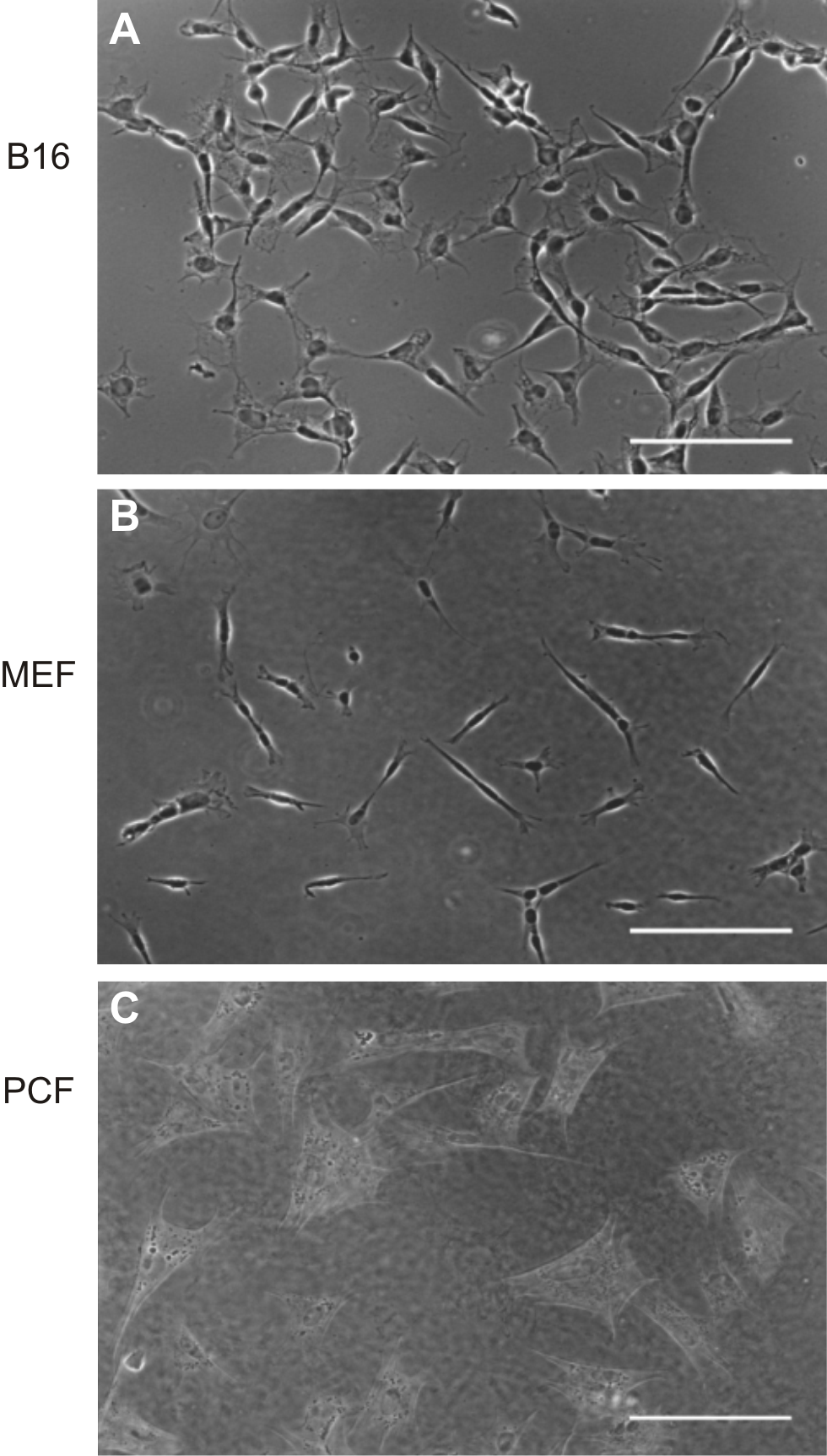

Supplement: Figure S1 — DIC images of the three cell types studied. Cells were seeded onto homogeneously fibronectin-functionalized glass cover slips and incubated for 2 hours before fixation. A) Mouse melanoma B16F1 cells, B) mouse embryonic fibroblast (MEF) cells and C) primary chick fibroblast (PCF) cells (scale bars = 100 µm). (TIF) [file pone.0054850.s001.tif]

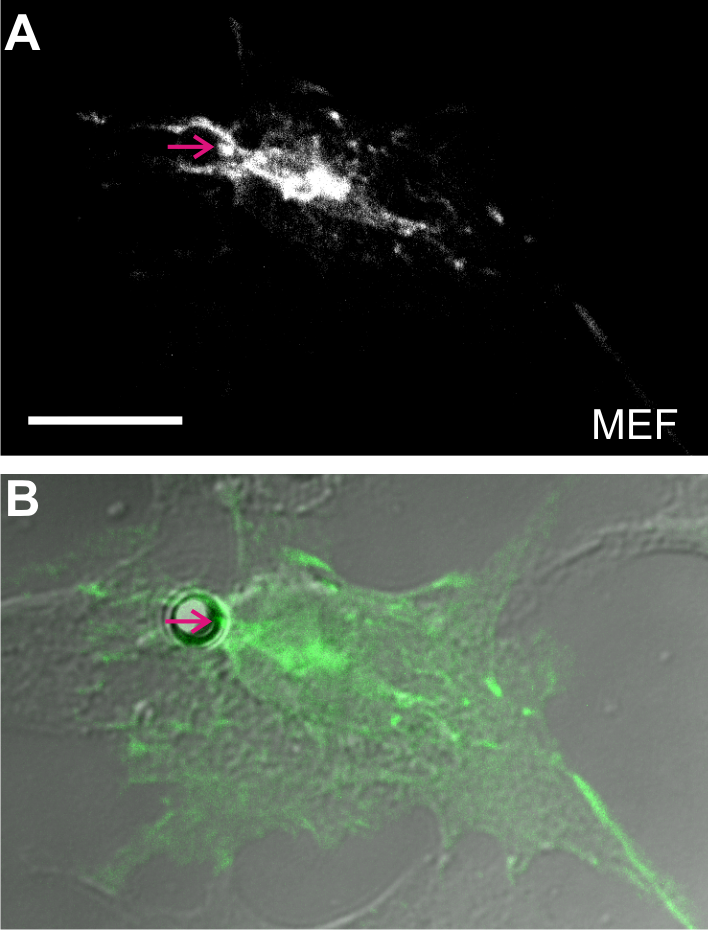

Supplement: Figure S2 — To test whether cells accepted the FN functionalized beads to invoke new adhesion sites on the surface, MEF cells were transfected to express a full length vinculin-GFP fusion protein. In figure S2 A) the accumulation of vinculin at the membrane/bead interface is depicted. The bead on the cellular leading edge did not experience any external force as it was not restrained by the optical forces of the laser trap. In about 50% of the examined cells a vinculin-GFP circle had formed around the bead, confirming the formation of adhesion sites at the contact area. B) Overlay of a DIC image with the fluorescent channel (scale bar = 10 µm). (TIF) [file pone.0054850.s002.tif]

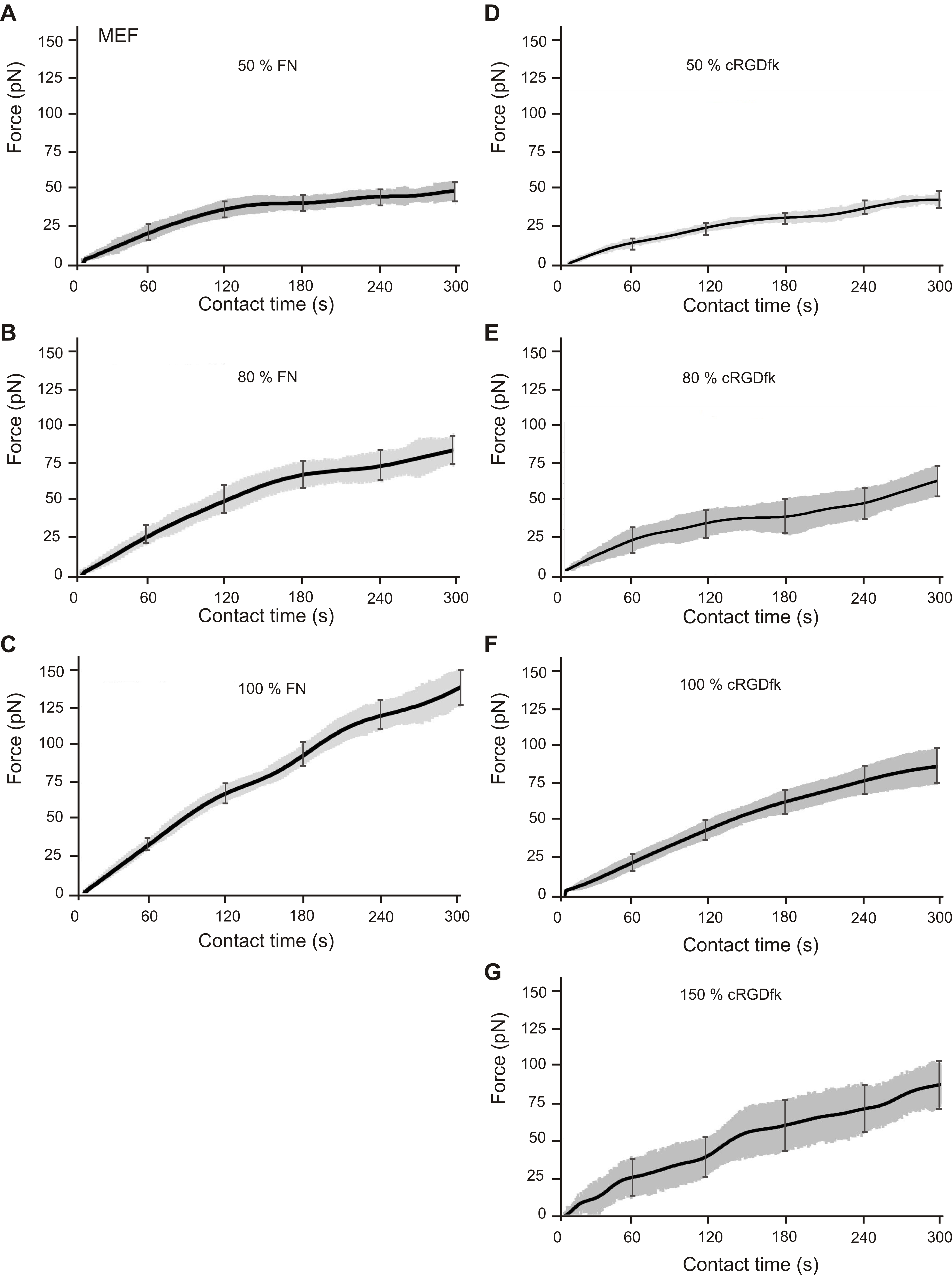

Supplement: Figure S3 — Averaged force-time curves for each surface density of FN and cRGDfk functionalized beads. A) to C) show the development of cellular traction force exertion onto FN-beads and D) to G) onto cRGDfk-beads (mean ± s.e.m; the s.e.m. is denoted in gray and the gray bars are error bars representing specific time points, N = 5, n = 8–13). (TIF) [file pone.0054850.s003.tif]
